# Shadow and extended shadow cost sharing associated to informal long-term care: the case of Spain

**DOI:** 10.1186/s13561-020-00272-1

**Published:** 2020-05-19

**Authors:** Raúl Del Pozo-Rubio, Pablo Moya-Martínez, Marta Ortega-Ortega, Juan Oliva-Moreno

**Affiliations:** 1 Department of Economics and Finance, University of Castilla-La Mancha, Avda, Los Alfares, 44 16.071, Cuenca, Spain; 2 Department of Applied Economics, Public Economics and Political Economy, Complutense University of Madrid, Campus de Somosaguas s/n. 28.223 Pozuelo de Alarcón, Madrid, Spain; 3 Department of Economics and Finance, University of Castilla-La Mancha, Calle San Pedro Mártir, 7, 45002 Toledo, Spain

**Keywords:** Informal care, Economic value, Long-term care, Spain, D63, I38, J14

## Abstract

**Background:**

A large part of the long-term care is provided by non-professional caregivers, generally without any monetary payment but a value economic of time invested. The economic relevance of informal caregivers has been recognized in Spain; however, public provision may still be scarce. The objective of this paper is to estimate the economic burden associated with informal long-term care that should assume the families through a new concept of cost sharing that consider opportunity costs of time provided by informal caregivers.

**Methods:**

The study sample includes all dependent adults in Spain. Socioeconomic information and the number of hours of informal care was collected through the Spanish Disability and Dependency Survey. The terms of shadow and extended shadow cost sharing were defined as the difference between the maximum potential amount of money that families could receive for the provision of informal care and the amount that actually they received and the value of informal care time with respect to the amount received, respectively.

**Results:**

53.87% of dependent persons received an economic benefit associated to informal care. The average weekly hours of care were 71.59 (92.62 without time restrictions). Shadow cost sharing amounted to, on average, two thirds, whereas the State financed the remaining third. In terms of extended shadow cost sharing, the State financed between 3% and 10% of informal care provided by caregivers.

**Conclusions:**

This study reveals the deficient support received for the provision of informal care in Spain. More than 90% of informal care time is not covered by the economic benefits that families receive from the State.

## Introduction

Long-term care (LTC) services are those services required by individuals who are unable to perform basic daily routines over a long time period [1]. In this sense, LTC service providers are faced with the challenge of providing personal care in the context of an aging population and increasingly changing social structure.

There is an evident difference in the number of LTC dependents in each country, ranging from less than 5% of the 65- to 74-year-old population in high-income countries to 50% in low- and middle-income countries [1, 2]. However, it has been estimated that, in high-income countries, the demand for LTC services among people of 80 years old and older will increase from 4% in 2010 to 10% in 2050 [1].

Currently, there are different models of LTC systems throughout Europe, with overall spending varying as a percentage of gross domestic product, coverage conditions, LTC baskets, financing sources (general taxes, social security contributions, private insurance, co-payments and direct payments for services) and the weight and relevance of informal care to total personal care [3].

In 2007, a new System for the Promotion of Personal Autonomy and Assistance for Persons in a Situation of Dependency (SAAD) was established in Spain through the approval of Act 39/2006 of 14 December (the Dependency Act, DA) [4], in order to attend to those people who are dependent on help with basic activities of the daily living. The DA acknowledged Spanish citizens’ universal entitlement to social services (eligibility being determined based on the level of dependency). This represented a large-scale structural change in the organization of LTC in Spain [5]. Although the DA was initially proposed as a normative change that would increase the provision of in-kind services (residential care, day/night care or home-help services), and would only consider the provision of economic benefits for informal care [6], the 2008 financial crisis and subsequent economic recession severely impacted the newly developed SAAD. First, the economic benefits for informal care became the primary benefit of the system. Undoubtedly, this was favoured given the lower associated cost per user compared to in-kind services. Second, in 2012, Spain faced a complex financial and economic crisis [7]. Thus, structural reform of the DA was enacted [8, 9], despite the previous reforms applied in 2010 [5, 10]. Among the major changes were a considerable reduction in cash benefits allocated to informal care, limits placed on qualifying conditions to access care and the renouncement of social contributions paid to informal caregivers by the State [11, 12].

Usually, co-payment is defined as a situation in which the cost of providing a service is shared between the user and the financing entity (usually the public or private insurer [13]). However, in the case of long-term care, a large part of them, according to some authors, up to 80% [14], is provided by non-professional caregivers, where there is no monetary payments but a value economic of time invested.

Much of the literature focuses on the effect of cost-sharing and out-of-pocket (OOP) payments in the accessibility of health care services. In some ways, cost sharing reduces the moral hazard in the consumption of unnecessary health care services. Unfortunately, cost sharing also deteriorates access to essential health care and adherence treatments, which in turn may lead to worse health outcomes and similar or higher health care costs [15–19].

One of the major concerns is the catastrophic financial risk incurred by households. Studies performed in different countries have explored the incidence of catastrophic risk resulting from OOP health care payments, which include cost sharing, spending as a whole. Most studies have focused on Asian (Vietnam [20, 21], the Western Balkans [22], Mongolia [23], Nepal [24], Iran [25], Thailand [26], Turkey [27] and China [28]) and African (the Middle East and North Africa [29] and Kenya [30]) countries. However, there are also studies from Latin America [31] and Brazil [32], as well as two recent studies conducted in Portugal [33] and Greece [34]. OOP payments have also been studied for specific subsamples, such as care for the chronically ill [35, 36] and disabled persons [37–39]. A recent systematic review concluded that when formal fees are introduced, protection against catastrophic health care payments is needed for the most vulnerable groups [40]; however, to our knowledge, no study has focused on payments allocated to long-term care services.

The objective of this study is to analyse the economic contribution and estimate the real economic burden of the dependent household that received informal care associated with LTC provided in Spain. To accomplish this, we adopt a double definition of cost-sharing, shadow cost sharing and extended shadow cost sharing (euros of 2017), exploring the differences depending on the level of dependence of the individual receiving care and the amount of time that care is provided.

## Materials and methods

### Data

We drew upon the Spanish Disability and Dependency Survey (SDDS) conducted by the Spanish National Statistics Institute [41]. This is the most recent representative survey of the disabled population living in Spanish households and includes information regarding the characteristics of personal care (i.e., number of care hours) and a household’s socio-demographic characteristics. The survey includes a total of 22,795 individuals with disabilities, representing 3.79 million people in population terms by means of elevation factors.

According to the study objectives, we selected people with dependency level I, II and III (*n* = 6523), older than 18 years old who received at least 1 h of informal care per week and had a reported household income (*n* = 360 no reported income), accounting for approximately one fifth of the original sample (*n* = 4794). The rest of the sample is people with disability, but they did not get the minimum score to be considered as person with at least mild level of dependency (level I) [42]. The DA defines three levels of dependency according to the level of severity: mild (level I), moderate (level II) and severe (level III) (see point 2.3). Inferences about the population from the sample results were made from population weights provided by the survey itself [43].

### Variables

#### Cost-sharing

The information regarding the costs of each dependency service and the amounts of money offered for the informal care by the public system were established in the structural reform of the 2012 DA which remain unchanged to date [8].

Specifically, the economic benefit provided by the State for informal care consists of a monthly allowance, adjusted by level of dependency, issued to the dependent person to be used to compensate the informal caregiver. This benefit is conditioned on compliance with certain restrictions that dependent person and informal caregiver must to be met and that we can find in the DA [8]. For example, once of the most important could be that the informal caregiver can only be the spouse and their relatives by consanguinity, affinity or adoption, up to the third degree of kinship, when they live together and dependent person is cared by them for at least 1 year before to the date of presentation of the application.

Several reduction factors are applied to the maximum amounts, which depend on the economic capacity of the beneficiary (composed of employment and self-employment income, capital income and wealth) and their level of dependence; thus, the final amount that the individual receives from the State is the *cash benefit for informal care* (CBIC). We have defined ***shadow cost sharing*** as the difference between the maximum amount that dependent person could receive, depending on their dependence level, and the amount of money that receives (CBIC). Additional file 1 describes the estimation of shadow cost sharing.

We define ***extended shadow cost sharing*** as the total amount of benefit that the caregiver should receive if all the time allotted to care was valued appropriately, calculated by subtracting the amount actually received (CBIC). Thus, the concept of extended shadow cost sharing factors the opportunity cost of caregiver’s time.

The time spent providing informal care is obtained from the recall method, i.e., questions collected in the SDDS. The recall method allows to measure the time dedicated to informal care when it is assumed that dependents take into account join production when they complete the questionnaire (for example, having a nap or watching TV and supervising after a care recipient with Alzheimer disease). Otherwise, the recall method will overestimate the time spent providing informal care [44, 45]. As we don’t know if survey respondents have taken into account the join production, a conservative approach was adopted for the primary analysis, and care provided was limited to a maximum of 16 h per day [46]. However, in the sensitivity analysis, that restriction was eliminated.

Several techniques are applied in the literature for valuing informal care time [45, 47, 48] highlighting the revealed preference methods and stated preference methods, among others. The difference between revealed and stated preference methods is mainly explained by the fact that the former uses responder’s preferences for nonmarket goods. The first method uses real life decision data, that is, the preferences are taken from informal caregivers’ decisions or from close substitutes in the market. Within stated preference methods, contingent valuation and conjoint analysis methods are included. However, the revealed preference methods are based on data from real choices that people decide. Two different approaches are included in this method; the opportunity cost and proxy good method.

The opportunity cost method (OCM) was used for the main analysis to value monetary assessment of care time. This approach values ​​informal care as the informal caregiver’s foregone benefit as a result of time spent providing informal care [45]. This method values the best alternatives that caregivers had to resign in order to provide the care. Following the methodology applied in other works [49, 50], caregivers were considered to invest their time in paid work, non-paid work (such as housekeeping or voluntary work) and leisure time. Given that in Spain, there are no recent estimations of the value associated with leisure time, in practical terms leisure and non-paid work time are valued using the same shadow price [51]. To estimate the value of informal care time, three groups were considered based on the information provided by each caregiver.

- Group 1 consists of those caregivers who were forced to leave their jobs to provide care services. Given the lack of available information regarding weekly work hours by caregivers before leaving the work force, full-time work was assumed (37.5 h/week). Care time was valued according to the average full-time wage per hour in Spain in 2017 disaggregated by regions [52]. The additional hours dedicated to informal care were valued at the minimum salary of a household employee in Spain in 2017, which was €4.42/h [53].
- Group 2 consists of those workers who reduced their working hours. Given the lack of information available and following the methods of other authors, it was assumed that the caregivers worked a total of 37.5 h/week, requiring a reduction of three working hours per day to provide care services [54]. The economic valuation of informal care hours, labour time reduced and additional time, was calculated as in Group 1.
- Group 3 consists of those caregivers who stated that they were not in the workforce or that they were not having problems balancing work with care. The time spent providing care in this case was valued using the minimum wage of a household employee in Spain in 2017 (€4.42/h) [53].

As part of the sensitivity analysis, two other valuation methods were used. First, the proxy good method (PGM) was used. PGM is a revealed preference method which assesses the time dedicated to informal care at the market price of the nearest substitute benefit [45]. This technique values the care provided considering how much it would cost to society or the family members if informal caregivers would disappear and, consequently, they had to be replaced at the labour market by a close substitute. To accomplish this, the cost of in-home service in Spain was used, calculated according to the workload and specific prices published by each community in 2016 and updated to 2017 [55]. Likewise, the contingent valuation method (CVM), which is the most commonly used stated preference method, was used. This approach evaluates the time of caregiving taking into consideration the caregivers’ well-being in a money metric, with compensation variation and equivalent variation for estimating the willingness to pay (WTP) and willingness to accept (WTA) for a hypothetical caregiving situation. WTP consists in estimating how much the responders are willing to pay at maximum in order to reduce 1 h of caregiving. Likewise, WTA consists in estimating how much the responders are willing to accept (to be compensated) at minimum in order to increase in one extra hour of care. To accomplish this, the monetary value of €6/h was used for WTA and the value of €3/h for WTP for 1 h of extra care [56]: both prices were updated to euros of 2017.

Therefore, four techniques have been used in this study (opportunity cost, proxy good, WTP and WTA) to assess the monetary value of the time of informal care. Other techniques, such as conjoint analysis or wellbeing method [57–59], have been excluded because there are no recent estimations regarding the time of informal care in Spain using these methods.

It is important to note that when the SAAD resolves that a person has the right to receive a benefit, according to the degree of dependence with which he has been classified, they can choose between receiving an in-kind service or an economic benefit. Shadow and extended shadow cost sharing were estimated by weighting the probability that the beneficiary would choose the economic benefit associated to informal care based on the existing statistical information by region and level of dependency [10] provided in previous studies [12, 60] (see Table A1 in Additional file 1).

### Dependency level

Dependent individuals are classified in one of the three levels of dependency defined by the DA according to the final sum of the value obtained from an official scale published in the Spanish Official Bulletin [42]: 0–24 points, no dependence; 25–49 points, level I or mild level; 50–74 points, level II or moderate level; and 75–100 points, level III or severe level. The official scale considers 47 daily tasks (eating and drinking, control of physical needs, bathing and hygiene, other physical care, dressing and undressing, maintaining one’s health, mobility, moving inside the home, moving outside the home and housework). The final score is the sum of the values of the tasks multiplied by the degree of the supervision required and the weight assigned to that activity. Although the survey used does not contain this official scale, the large number of questions included allows us to approximate the level of dependence as defined by the DA [50, 51].

### Scenarios of income

Original household income was categorized as follows: less than €500; €500–999; €1000–1499; €1500–1999; €2000–2499; €2500–2999; €3000–4999; €5000–6999; €7000–8999; and more than €9000. However, because the range is considerable in some categories, three economic capacity scenarios for the beneficiary were generated: in the first scenario, mark class were used (middle scenario), and in the second and third scenarios, as a sensitivity analysis, the lower end of the range (minimum scenario) and the upper end of the range (maximum scenario) were used, respectively.

The monthly household income of the beneficiaries was valued at 2017 euros using the consumer price index of Spain as an update factor [61].

All analyses were performed using STATA 13.0 statistical software (StataCorp LP, College Station, TX).

## Results

Tables A2 and A3 (Additional file 1) show the socio-demographic information of the dependent individuals in Spain who received at least 1 h of informal care (population-wise) and of the informal caregivers, respectively. An estimated total of 757,192 individuals are dependent, of which 30.70% are considered to have level I (mild) dependence; 40.38% have level II (moderate) dependence; and 28.92% have level III (severe) dependence. The dependent population has an average age of 74.46 years (standard deviation, SD: 17.30); they are predominantly females (66.3%), widowed (43.1%) and married (40.3%), with an incomplete basic education (illiterate or primary school incomplete) (62.5%), and retired (receiving earnings-related pension) (86.2%). The average monthly income is €1468.76 (SD: €1040.05).

Regarding the prevailing characteristics of caregivers, Table A2 shows they have an average age of 53.71 years old (SD: 13.17) and majority of them are women (73.3%) who are married (68.97%). Caregivers are characterized as having at least a basic education (37.43%), and their occupations are equally distributed between being a housewife (31.37%), retired (29.61%) and employed (27.16%). Note that 82.73% of caregivers reside in the home of the dependent person.

It is estimated that of the total population eligible to receive some type of benefit from the SAAD (depending on the probability of service selected), 53.87% choose the economic benefit associated to informal care (Table 1). The average number of weekly hours received for informal care, restricted to 16 h of daily care, ranges from 57.77 (SD: 37.43) hours per week for level I dependents to 86.07 (SD: 27.49) hours for level III dependent care, translating to a range that varies annually from 3003.85 (SD: 1946.16) hours for level I to 4475.48 (SD: 1429.67) hours for level III. Thus, the average for the three levels equals 3722.87 (SD: 1855.37) annual hours. When the 16 h daily maximum limit for calculable care is removed, the average values increase to 92.61 (SD: 56.86) hours per week and 4815.82 (SD: 2956.89) hours per year.

**Table 1:** Number of hours of informal care received by individuals with dependency needs in Spain

	People who receive an economic benefit for informal care		Informal care hours with max. 16 h/day		Informal care hours without restrictions	
			Weekly	Annual	Weekly	Annual
	Average (n)	%	Average (SD)	Average (SD)	Average (SD)	Average (SD)
Mild	232,473	52.18	57.77 (37.43)	3003.85 (1946.16)	71.76 (56.45)	3731.64 (2935.49)
Moderate	305,736	55.96	71.74 (35.37)	3730.53 (1839.21)	92.45 (56.48)	4807.47 (2937.00)
Severe	218,983	52.75	86.07 (27.49)	4475.48 (1429.67)	114.97 (48.80)	5978.44 (2537.60)
Total	757.192	53.87	71.59 (35.68)	3722.87 (1855.37)	92.61 (56.86)	4815.82 (2956.89)

Source: own elaboration from the Spanish Disability and Dependency Survey

*SD* Standard Deviation

Based on the hours of informal care received, the economic amount granted by the State to families who choose the economic benefit for informal care is estimated. Table 2 reveals, for different income scenarios, that the average benefit granted by the State amounts to approximately one third of the total funding in terms of shadow cost sharing, i.e., €1179.15 per year (ranging from €723.82 for level I and €1645.59 for level III). However, on average, this represents only 7.28% of the annual monetary value of informal extended care (extended shadow cost sharing) and 36.66% of shadow cost sharing. In other words, in terms of shadow cost sharing, the beneficiary contributes an average of €2036.99 annually (€1112.18 for level I and €3006.09 for level III), i.e., the remaining two thirds (63.34%) of the total benefit funding. This amount increases to €17,596.67 per year if the opportunity cost of the caregiver (extended shadow cost sharing) is considered, representing a financing of 92.72% of the service.

**Table 2:** Average and total annual amount of economic benefit granted by the State for economic benefit for informal care, including the associated shadow and extended shadow cost sharing

	Amount of benefit granted by the State		Shadow cost sharing			Extended shadow cost sharing (opportunity cost method)		
	Average	Standard Deviation	Average	Standard Deviation	%	Average	Standard Deviation	%
**Scenario 1**								
Mild	723.82	682.98	1112.18	747.22	60.58	14,096.85	11,770.98	95.12
Moderate	1187.07	1139.56	2038.41	1241.60	63.20	17,863.61	11,601.59	93.77
Severe	1645.59	1655.55	3006.09	1722.98	64.62	20,695.00	10,656.21	92.63
Total	1179.15	1259.16	2036.99	1476.03	63.34	17,596.67	11,702.68	92.72
**Scenario 2**								
Mild	976.81	813.46	859.19	814.96	46.80	13.848,32	11,729.96	93.41
Moderate	1609.61	1327.91	1615.87	1361.26	50.10	17,443.91	11.569.93	91.55
Severe	2252.92	1950.29	2398.76	1921.62	51.57	20,090.04	10,606.69	89.92
Total	1603.60	1501.60	1612.54	1538.84	50.14	17,172.51	11,634.85	91.46
**Scenario 3**								
Mild	455.79	564.05	1380.21	710.22	75.17	14,823.24	15,889.88	97.02
Moderate	735.73	971.67	2489.75	1159.03	77.19	18,031.41	14,801.03	96.08
Severe	1001.93	1391.86	3649.75	1591.07	78.46	20,986.66	14,685.90	95.44
Total	727.86	1041.36	2488.27	1477.88	77.37	17,914.20	15,301.67	96.10

Amounts in euros of 2017; Scenario 1 take for estimations average income, scenario 2 lower income range and scenario 3 upper income range. Source: own elaboration from the Spanish Disability and Dependency Survey

In scenario 2 (lower income range), there is evidence of an equal distribution in terms of the shadow cost sharing of the financial burden between the State (49.16%) and beneficiary (50.14%), equity that otherwise disappears in scenario 3 (upper income range), where the contribution of State is reduced (22.63%) and increases for the beneficiary (77.37%). However, in terms of extended shadow cost sharing, the contribution of the State ranges between 3.90% in scenario 3 and 8.54% in scenario 2, with the beneficiary assuming more than 90% of the cost of care.

**Fig. 1:**
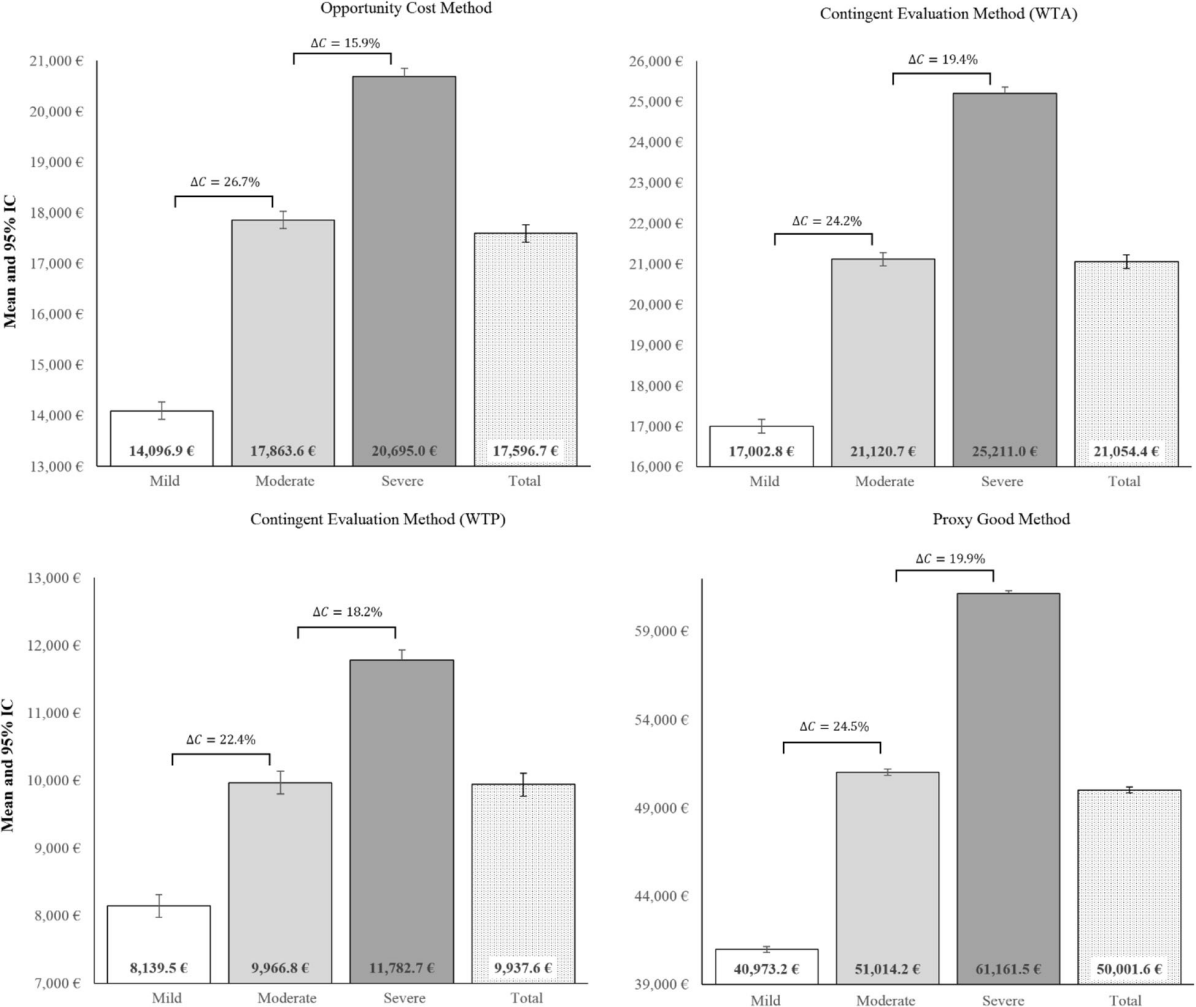
Sensitivity analysis [1]. Estimated annual monetary value of the extended shadow cost sharing of the economic benefit for informal care according to informal care assessment methods (**with restriction** in the number of daily hours of care). *∆C* refers to percent increment from one stage to another. Amounts in euros of 2017. Source: own elaboration from the Spanish Disability and Dependency Survey

**Fig. 2:**
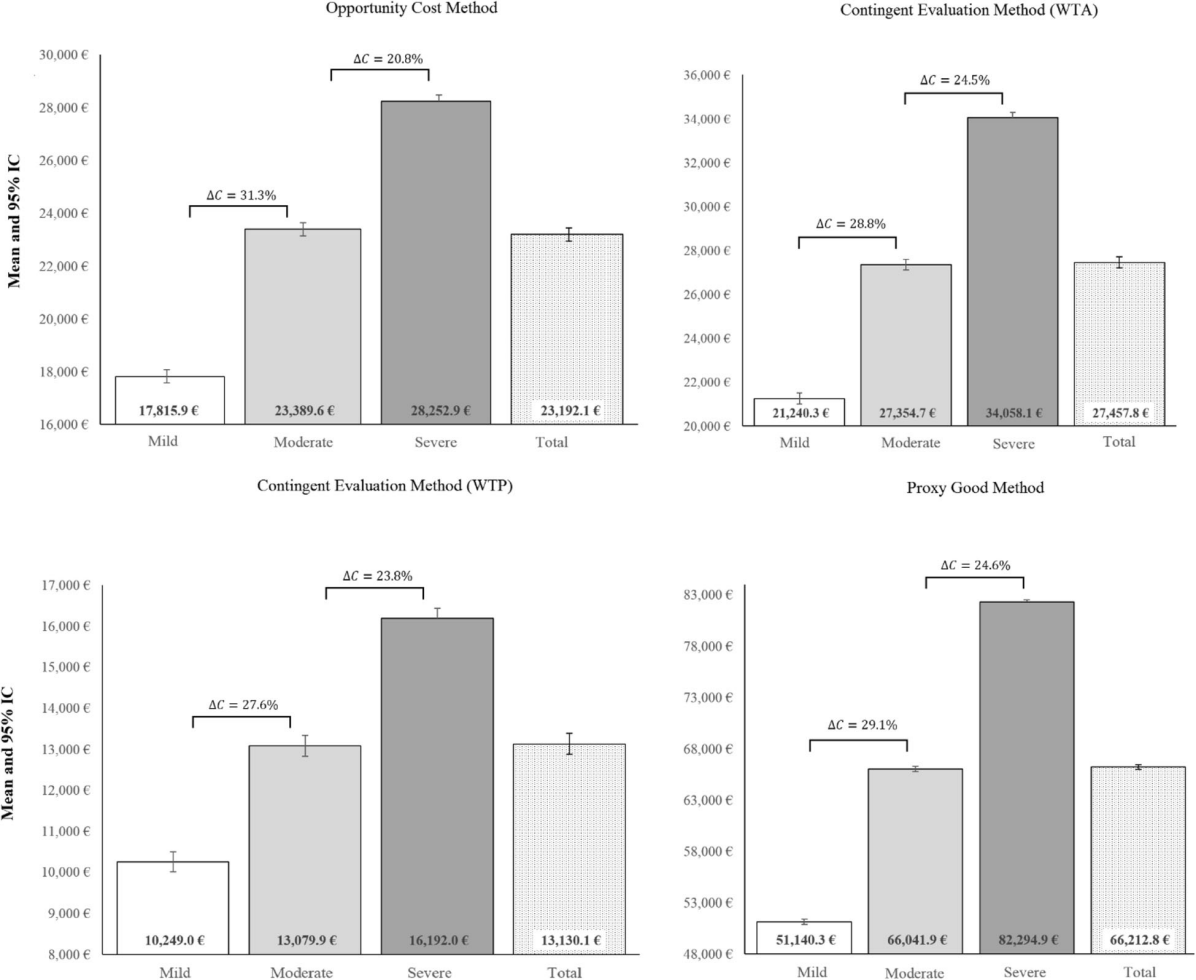
Sensitivity analysis [2]. Estimated annual monetary value of the extended shadow cost sharing of the economic benefit for informal care according to informal care assessment methods (**without restriction** in the number of daily hours of care). *∆C* refers to percent increment from one stage to another. Amounts in euros of 2017. Source: own elaboration from the Spanish Disability and Dependency Survey

Extended shadow cost sharing is also exhibited in Figs. 1 and 2, and Tables A4, A5 and A6 (see Additional file 1) where the sensitivity analysis shows the range of results. Scenario 1 (Table A4) shows that the average annual assessment of the extended shadow cost sharing per dependent, using the OCM, increases across the three levels when placing a limit (€23,192.05) on the number of hours worked, with significant differences that depend on the level of dependence (€17,815.89, €23,389.62 and €28,252.90, for levels I, II and III, respectively). PGM and WTA increase the amounts estimated by 290% and 20% compared to the amounts estimated using the OCM of up to €50,901.62 (€66,212.81 unrestricted) and €21,054.37 (€27,457.80 unrestricted) annually, respectively (scenario 1). Meanwhile, WTP reduces the amount estimated for OCM by 44% to €9937.61 (€13,130.07 unrestricted).

## Discussion

In summary, this study provides pragmatic information on the coverage of informal care by the DA and its implementation, finding that overall more than 90% of informal care time is not covered by the economic benefits that families receive from the State. This situation can result in critical social consequences for Spanish families, which, when taken in the context of a significant economic crisis, slow economic recovery and an inaccessible labour market, can lead to severe situations of social and financial catastrophe. In this sense, a recent study between 2006 and 2013 showed that multiple care strategies have become more common and care has become more externalised from the domestic domain [62], so we cannot expect the magnitude of the impact if DA had not been implemented in 2007. This could also suggest that the spouses, the principal informal caregivers, are receiving social service help and other workers who previously did not receive [62].

According to the literature, there is no an optimal relationship between formal and informal care equivalent for all people [63–65]. Different theories have analysed the need of utilization and combination of both types of care, formal and informal care. Supplementary Care Model postulates that most of the care is assumed by informal care, while formal care operates temporarily and circumstantially [66–68]. However, Complementary Care Model argues that formal care emerges when needs exceed the capabilities of informal care [67, 68]. Finally, in the Hierarchical Compensation Model, the informal caregiver establishes a ranking of preferences of activities to carry out (according to his availability), and formal caregiver emerges for the rest of activities [67–69]. Nowadays, literature suggests that the ideal composition and distribution of formal and informal care should depend on the type and severity of the limitations in the autonomy and needs of the person, the environment and family situation, as well as the availability of professional care resources.

Following our results, Spanish families bear a disproportional weight in the care of dependent people against the original aim of Spanish Dependency Act whose main goal was to offer formal services as a quasi-exclusive type of attention to dependent people. The main reason seems to be that 4 years after implementation, some Spanish regions became to grant eight out of ten benefits as cash benefit for informal care instead of in-kind services [70]. This means that in the coming years, public authorities must make an important effort in terms of providing professional services and support to family caregivers. Likewise, it is necessary to progress in strategies that coordinates in a more intense way, or even integrates, the formal and informal resources. In the case of Spain, it is critical the revision of the Dependency Act. The objective to be pursued would be the guarantee the well-being of caregivers and of the carers.

On the other hand, the choice of the technique for the assessment of informal care is not neutral, since it leads us to a very different estimated values, as indicated in previous studies [56]. Interestingly, while in the case of productivity losses there has been a very intense debate and there exists an extensive literature comparing the two main methods used [71, 72], in the case of informal care there is no such intense methodological discussion [48]. This is possibly due to the fact that, firstly, the researchers have focused on generating evidence that shows the relevance of informal care in studies of the cost of illnesses and its inclusion in economic evaluations [73]. Secondly, there is a wide research path in the field of informal care, of which the assessment of care time is only a (relevant) part [48]. And thirdly, the method chosen depends on the availability of appropriate data. Therefore, it would not be incorrect to point out that eclecticism is relevant when choosing the valuation technique. In any case, opportunity cost method prevails in the literature [46, 73] and a growing use of different methods is being recommended [46, 56].

The present work has several limitations. On the one hand, assignment probabilities of dependency benefits have been applied to estimate the amount corresponding to shadow and extended shadow cost sharing, given the absence of such data in the literature. Thus, a conservative perspective is maintained in the simulation because it is assumed that all eligible persons receive economic benefits for informal care. In fact, in the Spanish system, since its implementation in 2007, there have been significant lags between dependence degree ratings, the recognition of benefit rights (where appropriate) and a subsequent benefit allowance [74] resulting in a “dependence limbo” [5]. Specifically, in 2015, 35% of the population recognized as being dependent was entitled to receive benefits but was in limbo and did not receive any in kind or monetary benefit [75]. In practice, this implies that our results are conservative, and the current cost sharing should exceed our estimates. On the other hand, it should be noted that the SDDS, through which information related to socio-demographic characteristics has been obtained, dates back to 2008, whereas the calculation and “co-pay” amounts inherent to the economic benefit for informal care correspond to the structural reform of DA of 2012. The difference between the 4 years should influence the results only minimally because, first, at the international and local level, the Spanish population composing the majority of the dependent population (> 65 years old) experienced only a very slight increase from 2008 (2.40%) to 2012 (7.72%) [76]; second, individuals with disabilities are a segment of the population with changes in prevalence rates that are very slow and insignificant over time [12], elucidated by the comparison between the curves of 1999 and 2008, in which individuals with disabilities compose 6.2% and 6.5% of the population, and those with dependency compose 4.4% and 5.1%, respectively [77]. The third limitation is that the SDDS is a cross-sectional and not longitudinal data survey, which hamper to observe the evolution of informal care throughout the cycle of life of the people (for both, dependent people and informal caregivers). Last limitation is referred to the shadow price used to estimate the value of informal care using the opportunity cost approach, it is the same value for both types (leisure time and unpaid production time). As we mentioned in the methodological section, we used as proxy the minimum wage of household employees in Spain for both types because there is no information available regarding the real values.

Regarding to the “dependence limbo”, there is a second aspect of unmet needs for the informal care of individuals with disabilities who do not attain level I dependency. This study reveals that these individuals require a weekly average of 44 h of informal care, being in line with other researches carried out in the United States and Germany that reported around 50 h per week [78, 79]. The families of those individuals who are not eligible to receive benefits assume the total extended shadow cost sharing. In addition, our results support the overburden of the informal caregiver when the severity of the disease increases [51, 80], such as dementia [81], Alzheimer’s disease [82] or cancer [83, 84].

It should also be noted that our analysis does not consider other private expenses related to the care of dependents [85] (transport, adaptation of the home, professional care contracted by the family, etc.) or the medium- and long-term repercussions of other social opportunity costs (spillover effects) that are beyond the scope of our analysis. Thus, it should be emphasized that regardless of the method followed, the estimated monetary figures reflect the large amount of time spent by informal caregivers to care for dependents. This can translate into an immense burden for the caregivers, with important consequences to their health, work relationships and household finances [68]. Furthermore, although most studies tend to focus on the negative aspects of informal caregiving, there are other studies that show that caregiving can be a positive experience for the carer [48, 86–89], particularly in terms of psychosocial effects related to personal well-being and satisfaction with caring for another person [90–92]. In fact, the study of multidimensional factors that influence caregiver burden and satisfaction is a rising line of research.

## Conclusions

Despite the development of professional LTC programmes, informal care is a fundamental pillar in the care provided to individuals with limited autonomy. Although more prevalent in some countries than others for historical and cultural reasons, the existence of informal care is evident in any European LTC programme [93–95]. To date, informal care has been an “invisible” resource [96], and the support that these caregivers provide their families has been insufficiently recognized socially and economically, despite the significant burden and impact on the daily life of the caregiver and their quality of life related to health [48, 97]. However, the demographic and social changes that we are experiencing are modifying the social perception towards this resource of extraordinary value. Long-term care systems in Europe are moving towards mixed models, where responsibility for care is shared between the State and families [94]. More active policies of supporting carers (cash for care schemes and cash benefits, training, carers assessments and legislation) are growing in Europe taking us to a transition scenario that points to a professional or at least a formalization of informal care [98]. However, this process is occurring at different speeds across countries.

Several studies have focused on analysing the value of informal care provided to individuals with different diseases or varying levels of dependency, identifying the amount of informal care required to serve individuals with neurodegenerative, mental, cardio, cerebrovascular and rare diseases, among others [46, 99]. However, to our knowledge, none of these studies estimated the corresponding co-payment exclusively for the economic benefit designed for informal care, and an extended co-payment version has not been included based on the principle of the opportunity cost of time spent [45, 100–102]. Even in countries where cash benefits exist for the provision of informal care, such as in Germany, France and Sweden [94], any information about the benefit amounts or co-payments are not provided. The most approximate studies are focused on health expenditures for individuals with chronic diseases [35, 36] or disabilities [37–39]. The literature states that the annual OOP health care payments (including emergency, inpatient, outpatient and prescription drugs payments) for households with disabled members (those who most need LTC) is $1465 US or 1.29 times higher than households without disabled members. There is also a higher catastrophic incidence in individuals with a severe level of disability than moderate level [38] or $510 US for elderly households with chronic disease patients [37]. Both values translate to catastrophic rates (when OOP payments exceed a threshold – 40% is most common – or a certain percentage of income minus food expenditure [20, 103], which equal ratios of 11.5% for disabled persons and 30.57% and 22.03% for rural and urban households with chronic disease patients, respectively.

Our results show that families assume a significant burden from the provision of informal care: two thirds of shadow cost sharing are estimated in the middle-income scenario. Similarly, there is an extraordinary sensitivity in the results regarding economic capacity: a percentage of shadow cost sharing exists, which ranges from 50% (minimum-income scenario) to 75% (maximum-income scenario). At any rate, when economic capacity exceeds €1610 monthly, the CBIC is zero, and shadow and extended shadow cost sharing is 100%. However, extended shadow cost sharing exposes the financial aspect of the provision designed for informal care as it relates to the economic value of the provider’s time. The CBIC covers on average 6% of the assessed total informal care needs (range 3.90%–8.54%). According to the OCM and WTA, the results are very similar to each other, with the provision covering approximately 6.5%.

However, the number of weekly hours obtained for all individuals with a dependency (71.59) aligns with the results in Spain [104] and from a recent systematic review of informal care [46], highlighting the intense workload of southern European countries compared to other areas in the world, the former of which provide twice the number of care hours of northern or central European countries, North America, Asia, Oceania and medium and low income countries.

Future lines of research arise from the results of the present study. Firstly, it would be interesting to valuate informal care differences between regions in terms of equity (differences in costs and services supply) and catastrophism (derivate of shadow and extend shadow cost sharing). Secondly, to assess the economic impact of the replacement of informal care to formal care, in the case that informal care would disappear. Thirdly, to evaluate the opportunity cost and level of involvement of informal caregiver differentiating between a close relative or other figure (for example, using the shadow and extended shadow cost sharing). Finally, it would be relevant to analyse potential differences between rural and urban regions, given the depopulation trend that Spain is suffering.

Our results may be relevant for the design of social policies that recognize and focus on the informal care of individuals with disabilities or dependency needs. In fact, demographics and social dynamics suggest that an important component of the informal care that is being provided today will be unsustainable in the future and should be replaced by professional care [64, 105, 106]. Therefore, the estimated values of the extended shadow cost sharing not only reveal the burden borne by families today but also represent a forecast of future demand for professional services [64, 106–108], which will require private or public financing and, considering the high figures of time of care, a reorganizations of professional and informal webs of services. Thus, the development of care policies for dependents must better coordinate health, social (professional) and family (informal) resources to achieve their efficient and equitable use that translates into improvements in the overall wellbeing, not only of the people receiving care but also of the caregivers. This involves making informal care “visible”, improving its social recognition and supporting caregivers by providing training and administrative support, informing them of their rights and the professional services from which they can benefit, including respite services and other support elements [109, 110].

## Supplementary information

**Additional file 1.** Estimation of the shadow cost sharing of informal care and sociodemographic description.

## Data Availability

Available in: http://www.ine.es/dyngs/INEbase/es/operacion.htm?c=Estadistica_C&cid=1254736176782&menu=resultados&secc=1254736195313&idp=1254735573175, and please contact author for data requests.

## Abbreviations

LTC: Long-term care
SAAD: System for the Promotion of Personal Autonomy and Assistance for Persons in a Situation of Dependency
DA: Dependency Act
OOP: Out-of-pocket
SDDS: Spanish Disability and Dependency Survey
CBIC: Cash benefit for informal care
OCM: Opportunity cost method
PGM: Proxy good method
CVM: Contingent valuation method
WTA: Willingness to accept
WTP: Willingness to pay
