# Supplementary material for: Shadow and extended shadow cost sharing associated to informal long-term care: the case of Spain
Source: Health Econ Rev. 2020 May 19;10:12. doi: 10.1186/s13561-020-00272-1 (PMC7236927; doi:10.1186/s13561-020-00272-1)
Supplement: Supplementary file 1 — Additional file 1. Estimation of the shadow cost sharing of informal care and sociodemographic description. [file 13561_2020_272_MOESM1_ESM.docx]

**Appendix I**

*Estimation of the shadow cost sharing of informal care*

As previously mentioned, to estimate the shadow cost sharing, it is necessary to subtract the amount that the individual actually receives from the maximum amount that the State attributes to a certain level of dependence.

For the estimation of the latter, the State provides the following equation for each subject *j*:

$q_{j}=1.33P_{j}-\frac{0.44P_{j}B_{j}}{I}$ (1)

where *P_j_* is the maximum amount of the economic benefit for informal care and for the individual *j*, *B_j_* is the economic capacity of the beneficiary (composed of employment and self-employment income, capital income and wealth), and *I* is the public indicator of multiple effect income.

The maximum amount of *P_j_* depends on the level of dependence of the individual, with €153, €268.79 and €387.64 being the monthly amount for dependency levels I, II and III, respectively [35].

*B_j_* is defined as the full income, which includes employment, self-employment and capital income, as well as patrimony [35]. However, according to the available information, we used household monthly income as a proxy variable for economic capacity.

Finally, *I* is a monetary value used in the Spanish economy as a reference for the granting of assistance, grants, subsidies or unemployment benefits, among other allowances, and is set annually by lawmakers.

**Appendix II**

**Table A1**. Discrete multinomial probability distribution of economic benefit for informal disaggregated by level of dependency, according to place of residence*.

|  | Cash benefit for family care and help to support non-professional caregivers (informal care) | | |
| --- | --- | --- | --- |
|  | Level I | Level II | Level III |
| Andalusia | 53,84% | 56.67% | 59.29% |
| Aragon | 72,81% | 70.45% | 55.66% |
| Asturias | 56,02% | 56.75% | 43.79% |
| Balearic Islands | 83,10% | 80.75% | 67.03% |
| Canary Islands | 0,28% | 33.36% | 56.76% |
| Cantabria | 71,46% | 71.75% | 58.87% |
| Castile Leon | 47,00% | 47.54% | 41.00% |
| Castilla-La Mancha | 62,84% | 63.72% | 57.57% |
| Catalonia | 71,68% | 69.55% | 56.80% |
| Valencia | 41,87% | 47.80% | 51.09% |
| Extremadura | 53,91% | 54.43% | 40.56% |
| Galicia | 46,02% | 51.90% | 56.50% |
| Community of Madrid | 25,89% | 24.23% | 20.58% |
| Region of Murcia | 82,48% | 82.39% | 81.10% |
| Foral Community of Navarre | 81,64% | 71.31% | 52.58% |
| Basque Country | 53,68% | 54.33% | 45.53% |
| Rioja | 58,54% | 58.23% | 50.19% |
| Ceuta and Melilla | 75,78% | 76.25% | 74.25% |
| Total Spain | 57,49% | 58.00% | 52.69% |

Source: Statistics Service of the Attached General Department of Value, Quality and Evaluation of the System for Autonomy and Dependence Care

**Table A2.** Sociodemographic characteristics of dependent individuals who receive at least one weekly hour of informal care.

|  | | **Mild dependence**  **(level I)** |  | **Moderate**  **dependence**  **(level II)** |  | **Severe dependence**  **(level IIII)** |  | **Total** |
| --- | --- | --- | --- | --- | --- | --- | --- | --- |
| N | | 232.473 |  | 305.736 |  | 218.983 |  | 757.192 |
| Age (Mean)  (S.D.) | | 72.60  (16.74) |  | 74.22  (17.34) |  | 76.79  (17.47) |  | 74.46 (17.30) |
| Gender | |  |  |  |  |  |  |  |
|  | Male | 33.60 |  | 35.1 |  | 31.8 |  | 33,7 |
|  | Female | 66.40 |  | 64.9 |  | 68.2 |  | 66,3 |
| Marital status | |  |  |  |  |  |  |  |
|  | Single | 13.50 |  | 15.4 |  | 15.8 |  | 14,9 |
|  | Married | 45.70 |  | 40.1 |  | 34.8 |  | 40,3 |
|  | Widowed | 38.70 |  | 43.1 |  | 47.7 |  | 43,1 |
|  | Separated/divorced | 2.10 |  | 1.4 |  | 1.6 |  | 1,7 |
| Educational level | |  |  |  |  |  |  |  |
|  | Illiterate or primary school incomplete | 59.4 |  | 62.0 |  | 66.3 |  | 62,5 |
|  | Primary or equivalent | 24.2 |  | 23.4 |  | 23.5 |  | 23.7 |
|  | Secondary school/middle level profesional | 12.2 |  | 10.3 |  | 7.1 |  | 9.9 |
|  | University degree or equivalent | 4.2 |  | 4.3 |  | 3.1 |  | 3,9 |
| Activity status | |  |  |  |  |  |  |  |
|  | Employed | 3.0 |  | 1.3 |  | 0.5 |  | 1,6 |
|  | Unemployed | 1.2 |  | 0.5 |  | 0.5 |  | 0,7 |
|  | Receiving earnings-related pension | 82.7 |  | 87.9 |  | 87.6 |  | 86,2 |
|  | Other situtation | 13.1 |  | 10.3 |  | 11.5 |  | 11,5 |
| Dependency score* (Mean)  (S.D.) | | 42.25  (4.31) |  | 62.01  (7.30) |  | 86.50  (7.56) |  | 63.02 (18.33) |
| Monthly income household** (Mean)  (S.D.) | | 1,434.64  (1,032.96) |  | 1,472.47  (1,041.89) |  | 1,494.63  (1,044.20) |  | 1,468.76  (1,040.05) |

S.D.: Standard Deviation; * points; **Euros of 2017. Source: own elaboration from the Spanish Disability and Dependency Survey.

**Table A3.** Sociodemographic characteristics of principal informal caregiver of dependent individuals.

|  |  | **Mild dependence**  **(level I)** |  | **Moderate**  **dependence**  **(level II)** |  | **Severe dependence**  **(level IIII)** |  | **Total** |
| --- | --- | --- | --- | --- | --- | --- | --- | --- |
| Age (Mean)  (S.D.) | | 53.45  (13.59) |  | 53.56  (12.98) |  | 54.35  (13.03) |  | 53.71  (13.17) |
| Gender | |  |  |  |  |  |  |  |
|  | Male | 28.05 |  | 26.95 |  | 24.75 |  | 26,67 |
|  | Female | 71.95 |  | 73.05 |  | 75.25 |  | 73,33 |
| Marital status | |  |  |  |  |  |  |  |
|  | Single | 17.07 |  | 18.68 |  | 17.17 |  | 17,74 |
|  | Married | 70.00 |  | 68.80 |  | 68.06 |  | 68,97 |
|  | Widowed | 7.21 |  | 6.56 |  | 8.71 |  | 7,37 |
|  | Separated/divorced | 5.73 |  | 5.96 |  | 6.06 |  | 5,92 |
| Educational level | |  |  |  |  |  |  |  |
|  | Illiterate or primary school incomplete | 27.70 |  | 28.89 |  | 27.13 |  | 28,01 |
|  | Primary or equivalent | 37.96 |  | 36.09 |  | 38.72 |  | 37,43 |
|  | Secondary school/middle level profesional | 25.34 |  | 26.99 |  | 25.69 |  | 26,09 |
|  | University degree or equivalent | 8.89 |  | 8.03 |  | 8.47 |  | 8,43 |
| Nationality | |  |  |  |  |  |  |  |
|  | Spanish | 96.62 |  | 96.81 |  | 96.36 |  | 96,62 |
|  | Foreign | 2.93 |  | 2.85 |  | 3.38 |  | 3,02 |
|  | Spanish and other | 0.45 |  | 0.34 |  | 0.26 |  | 0,35 |
| Activity status | |  |  |  |  |  |  |  |
|  | Employed | 29.59 |  | 28.86 |  | 22.03 |  | 27,16 |
|  | Unempolyed | 7.97 |  | 9.92 |  | 9.08 |  | 9,07 |
|  | Receiving earnings-related pension | 29.75 |  | 28.35 |  | 31.23 |  | 29,61 |
|  | Housewife | 29.99 |  | 30.23 |  | 34.51 |  | 31,37 |
|  | Incapable of work | 0.28 |  | 0.56 |  | 0.62 |  | 0,49 |
|  | Student | 0.80 |  | 0.31 |  | 0.45 |  | 0,50 |
|  | Other situation | 1.64 |  | 1.62 |  | 2.00 |  | 1,73 |
| Live in the dependent’s home | | 78.44 |  | 82.72 |  | 87.11 |  | 82.73 |

S.D.: Standard Deviation. Source: own elaboration from the Spanish Disability and Dependency Survey.

**Table A4.** Sensitivity analysis. Scenario 1 for estimated annual monetary value of the extended shadow cost sharing of the economic benefit for informal care according to informal care assessment methods with and without restriction in the number of daily hours of care.

|  |  | Mild | | |  | Moderate | | |  | Severe | | |  | Total | | |
| --- | --- | --- | --- | --- | --- | --- | --- | --- | --- | --- | --- | --- | --- | --- | --- | --- |
|  |  | Mean | SD | % |  | Mean | SD | % |  | Mean | SD | % |  | Mean | SD | % |
| Opportunity Cost Method | |  |  |  |  |  |  |  |  |  |  |  |  |  |  |  |
|  | Restricted hours | 14,096.85 | 11,770.98 | 95.12 |  | 17,863.61 | 11,601.59 | 93.77 |  | 20,695.00 | 10,656.21 | 92.63 |  | 17,596.67 | 11,702.68 | 92.72 |
|  | Unrestricted hours | 17,815.89 | 17,121.65 | 96.10 |  | 23,389.62 | 17,252.29 | 95.17 |  | 28,252.90 | 16,544.54 | 94.50 |  | 23,192.05 | 17,516.04 | 95.16 |
| Proxy Good Method | |  |  |  |  |  |  |  |  |  |  |  |  |  |  |  |
|  | Restricted hours | 40,973.22 | 32,555.05 | 98.26 |  | 51,014.23 | 31,178.43 | 97.73 |  | 61,161.47 | 27,766.78 | 97.38 |  | 50,001.62 | 31,722.73 | 97.74 |
|  | Unrestricted hours | 51,140.26 | 47,374.46 | 98.60 |  | 66,041.92 | 47,537.74 | 98.23 |  | 82,294.85 | 44,605.75 | 98.04 |  | 66,212.81 | 48,272.00 | 98.25 |
| Contingent Valuation (WTP) | |  |  |  |  |  |  |  |  |  |  |  |  |  |  |  |
|  | Restricted hours | 8,139.51 | 6,589.70 | 91.83 |  | 9,966.82 | 6,239.19 | 89.36 |  | 11,782.69 | 5,428.57 | 87.75 |  | 9,937.61 | 6,304.34 | 89.39 |
|  | Unrestricted hours | 10,248.99 | 9,573.08 | 83.40 |  | 13,079.88 | 9,265.44 | 91.68 |  | 16,192.04 | 8,579.65 | 90.77 |  | 13,130.07 | 9,451.54 | 91.76 |
| Contingent Valuation (WTA) | |  |  |  |  |  |  |  |  |  |  |  |  |  |  |  |
|  | Restricted hours | 17,002.84 | 13,195.91 | 95.92 |  | 21,120.70 | 12,400.35 | 94.68 |  | 25,210.97 | 10,702.47 | 93.87 |  | 21,054.37 | 12,629.36 | 94.70 |
|  | Unrestricted hours | 21,240.34 | 19,143.88 | 96.70 |  | 27,354.66 | 18,474.79 | 95.84 |  | 34,058.07 | 17,085.94 | 95.39 |  | 27,457.80 | 18,948.19 | 95.88 |

Scenario 1 use average income range for estimations; Amounts in euros of 2017; Restricted hours employ a restriction in maximum hours of informal care of 16. Source: own elaboration from the Spanish Disability and Dependency Survey.

**Table A5.** Sensitivity analysis. Scenario 2 for estimated annual monetary value of the extended shadow cost sharing of the economic benefit for informal care according to informal care assessment methods with and without restriction in the number of daily hours of care.

|  |  | Mild | | |  | Moderate | | |  | Severe | | |  | Total | | |
| --- | --- | --- | --- | --- | --- | --- | --- | --- | --- | --- | --- | --- | --- | --- | --- | --- |
|  |  | Mean | SD | % |  | Mean | SD | % |  | Mean | SD | % |  | Mean | SD | % |
| Opportunity Cost Method | |  |  |  |  |  |  |  |  |  |  |  |  |  |  |  |
|  | Restricted hours | 13,848.32 | 11,729.96 | 93.41 |  | 17,443.91 | 11.569.93 | 91.55 |  | 20,090.04 | 10,606.69 | 89.92 |  | 17,172.51 | 11,634.85 | 91.46 |
|  | Unrestricted hours | 17,567.36 | 17,080.06 | 94.73 |  | 22,969.92 | 17,213.39 | 93.45 |  | 27,647.94 | 16,480.04 | 92.47 |  | 22,767.88 | 17,439.21 | 93.42 |
| Proxy Good Method | |  |  |  |  |  |  |  |  |  |  |  |  |  |  |  |
|  | Restricted hours | 40,720.23 | 32,510.03 | 97.66 |  | 50,591.68 | 31,125.32 | 96.92 |  | 60,554.13 | 27,699.25 | 96.41 |  | 50,477.17 | 31,636.72 | 96.92 |
|  | Unrestricted hours | 50,887.27 | 47,330.85 | 98.12 |  | 65,619.38 | 47,484.13 | 97.61 |  | 81,687.51 | 44,535.64 | 97.32 |  | 65,788.37 | 48,184.88 | 97.62 |
| Contingent Valuation (WTP) | |  |  |  |  |  |  |  |  |  |  |  |  |  |  |  |
|  | Restricted hours | 7,886.52 | 6,554.41 | 88.98 |  | 9,544.27 | 6,217.81 | 85.57 |  | 11,175.35 | 5,421.24 | 83.22 |  | 9,513.16 | 6,253.22 | 85.57 |
|  | Unrestricted hours | 10,248.99 | 9,573.08 | 91.30 |  | 13,079.88 | 9,265.44 | 89.04 |  | 16,192.04 | 8,579.65 | 87.79 |  | 13,130.07 | 9,451.54 | 89.12 |
| Contingent Valuation (WTA) | |  |  |  |  |  |  |  |  |  |  |  |  |  |  |  |
|  | Restricted hours | 16,749.85 | 13,153.30 | 94.49 |  | 20,698.16 | 12,360.06 | 92.78 |  | 24,603.63 | 10,645.24 | 91.61 |  | 20,629.92 | 12,551.86 | 92.79 |
|  | Unrestricted hours | 21,240.34 | 19,143.88 | 95.60 |  | 27,354.66 | 18,474.79 | 94.44 |  | 34,058.07 | 17,085.94 | 93.80 |  | 27,457.07 | 17,085.94 | 94.48 |

Scenario 2 use lower income range for estimations; Amounts in euros of 2017; Restricted hours employ a restriction in maximum hours of informal care of 16. Source: own elaboration from the Spanish Disability and Dependency Survey.

**Table A6.** Sensitivity analysis. Scenario 3 for estimated annual monetary value of the extended shadow cost sharing of the economic benefit for informal care according to informal care assessment methods with and without restriction in the number of daily hours of care.

|  |  | Mild | | |  | Moderate | | |  | Severe | | |  | Total | | |
| --- | --- | --- | --- | --- | --- | --- | --- | --- | --- | --- | --- | --- | --- | --- | --- | --- |
|  |  | Mean | SD | % |  | Mean | SD | % |  | Mean | SD | % |  | Mean | SD | % |
| Opportunity Cost Method | |  |  |  |  |  |  |  |  |  |  |  |  |  |  |  |
|  | Restricted hours | 14,823.24 | 15,889.88 | 97.02 |  | 18,031.41 | 14,801.03 | 96.08 |  | 20,986.66 | 14,685.90 | 95.44 |  | 17,914.20 | 15,301.67 | 96.10 |
|  | Unrestricted hours | 18,076.48 | 17,160.19 | 97.54 |  | 23,832.79 | 17,290.67 | 97.01 |  | 28,892.46 | 16,616.99 | 96.65 |  | 23,639.63 | 17,596.90 | 97.01 |
| Proxy Good Method | |  |  |  |  |  |  |  |  |  |  |  |  |  |  |  |
|  | Restricted hours | 41,241.25 | 32,595.72 | 97.54 |  | 51,465.57 | 31,226.03 | 97.01 |  | 61,805.13 | 27,838.47 | 96.65 |  | 51,352.91 | 31,808.73 | 97.01 |
|  | Unrestricted hours | 51,408.28 | 47,412.91 | 98.91 |  | 66,493.26 | 47,584.97 | 98.59 |  | 82,938.51 | 44,677.84 | 98.40 |  | 66,664.10 | 48,357.85 | 98.60 |
| Contingent Valuation (WTP) | |  |  |  |  |  |  |  |  |  |  |  |  |  |  |  |
|  | Restricted hours | 8,407.54 | 6,623.83 | 94.86 |  | 10,418.15 | 6,263.07 | 93.40 |  | 12,426.35 | 5,461.60 | 92.54 |  | 10,388.90 | 6,366.97 | 93.45 |
|  | Unrestricted hours | 10,523.49 | 9,596.55 | 95.85 |  | 13,539.02 | 9,286.21 | 94.85 |  | 16,853.36 | 8,606.43 | 94.39 |  | 13,592.25 | 9,510.10 | 94.92 |
| Contingent Valuation (WTA) | |  |  |  |  |  |  |  |  |  |  |  |  |  |  |  |
|  | Restricted hours | 17,270.87 | 13,235.64 | 97.43 |  | 21,572.04 | 12,438.66 | 96.70 |  | 25,854.63 | 10,773.39 | 96.27 |  | 21,505.66 | 12,711.76 | 96.73 |
|  | Unrestricted hours | 21,514.84 | 19,170.84 | 97.93 |  | 27,813.80 | 18,504.80 | 97.42 |  | 34,719.39 | 17,134.25 | 97.20 |  | 27,919.99 | 19,019.10 | 97.46 |

Scenario 3 use upper income range for estimations; Amounts in euros of 2017; Restricted hours employ a restriction in maximum hours of informal care of 16. Source: own elaboration from the Spanish Disability and Dependency Survey.
